# Consumption of Added Sugars by Rural Residents of Southwest Virginia

**DOI:** 10.13023/jah.0203.07

**Published:** 2020-07-19

**Authors:** Maryam Yuhas, Valisa Hedrick, Jamie Zoellner

**Affiliations:** University of Virginia; Virginia Tech; University of Virginia

**Keywords:** Appalachia, rural health, nutrition, sugar consumption, consumer health, sugar-sweetened beverage, rural populations

## Abstract

**Introduction:**

Nationally, rural residents have high consumption of added sugars, yet the top sources have not been explored. Characterizing added-sugar intake in high sugar-sweetened beverage (SSB) consumers in rural areas is an important step to help inform interventions and policies.

**Purpose:**

The objective of this study was to explore the top food and beverage sources of added sugar and to examine variations by sociodemographic characteristics.

**Methods:**

This cross-sectional study analyzed data from a randomized-controlled trial to reduce SSB in eight rural Appalachian counties. Data were obtained from baseline demographic surveys and three 24-hour dietary recalls. Dietary analyses included deriving AS grams and percentage of total energy intake from added sugar from individual food categories.

**Results:**

This study had 301 participants, of which 93% were White (non-Hispanic), 81% were female, 49% were aged 35 to 54 years, 43% had an income of ≤$14,000, 33% had low health literacy, and 32% had < college education. Males and those with an income of ≤$14,000 had significantly higher consumption of added sugar. Added sugar contributed to 21% of total energy intake. The top source of added sugar was soda. SSB contributed to 66% of added sugar and 14% of total energy intake. Within SSB, soda contributed to 40% of added sugar, and 8% of total energy intake. Cola and citrus flavored drinks were the main varieties consumed.

**Implications:**

Study findings can be used to adapt evidence-based interventions to reflect commonly consumed food and beverages and help inform food- and beverage-based dietary guidelines and policies specific to rural populations.

## INTRODUCTION

Excessive added sugar consumption is a nutritional determinant that influences high chronic disease rates observed across the U.S. Specifically, increased consumption of foods with added sugar is related to a higher risk for cardiovascular disease, obesity, Type-2 diabetes, dental carries, and cancer.^1^–^5^ The 2015–2020 Dietary Guidelines for Americans recommends limiting calories from added sugar to 10% or less of daily intake.^6^ Nationally, added-sugar intake accounts for 14% of total energy intake in the adult diet, primarily coming from soda, candy and grain-based desserts (e.g., cookies, cakes).^7^,^8^ Demographic factors such as being male, non-Hispanic black, and having lower income, education and health literacy have been found to be associated with higher intake of added-sugar foods.^9^,^10^

Unfortunately, information about added-sugar intake and specific food sources are lacking in areas of U.S that are disproportionately affected by chronic diseases influenced by the over consumption of added sugar, such as rural regions.^11^,^12^ Likewise, there are substantially fewer evidence-based interventions that target added sugar in rural regions when compared to other areas.^13^ Understanding the sources of added sugar in rural diets can help increase the cultural relevancy of interventions (i.e. focusing on food and drinks that are consumed most often) and inform policies targeting the most harmful sources of added-sugar intake in this region.

One such rural setting is the Appalachian area of Southwest Virginia. Appalachia is a region consisting of mostly rural counties where deaths from added sugar associated diseases (i.e., diabetes, heart disease, cancer, and stroke) and prevalence of obesity and poor oral health are higher compared to non-Appalachian residents.^14^,^15^ added-sugar intake in this region contributes to around 21% of total energy intake which is 7% higher than national intake and more than twice the recommended amounts.^6^,^16^ Within the added-sugar group, sugar-sweetened beverage (SSB) consumption also been found to be a pervasive problem in this region. One regional study found that residents in southwest Virginia were consuming 457 calories of SSB per day, more than three times the national intake.^17^,^18^ Given the disparities in added-sugar–related diseases and added-sugar intake in the Appalachian area of Southwest Virginia, understanding added-sugar food and beverage sources is imperative.

This study is a secondary data analysis of Talking Health, a randomized control trial in southwest Virginia that had a primary aim of reducing SSB intake.^19^ This trial compared the effectiveness of an intervention targeting SSB (SIPsmartER) against a matched contact, physical-activity based intervention (MoveMore). At baseline, SSB intake in this sample was 496 kcals/day. SIPsmartER was successful in reducing SSB intake by 227 kcals/day from baseline to 6 months, compared to 53 kcals/day in the MoveMore group.^19^ Data from the Talking Health trial has also been used to understand other dietary patterns in this rural population. For example, Hedrick and colleagues found that participants at baseline had an average energy intake of 1871 kcals/day, of which 21% were added sugar.^16^ However, the specific food and beverage sources consumed and their relative contribution to total added sugar and energy intake have yet to be explored. Therefore, the objectives of this study are to (1) explore the top food and beverage sources of added sugar and their contribution to total added sugar and energy intake and (2) examine variations across sociodemographic characteristics.

## METHODS

### Study Design and Participants

This study is a secondary analysis of cross-sectional data collected at baseline from the Talking Health trial.^20^ Talking Health was a randomized-controlled, community-based trial that occurred in eight rural counties across Southwest Virginia. These counties are federally designated as medically underserved, and have a rurality status of 6.1 ± 2.5 out of 9 on the Rural–Urban Continuum Codes (9 = very rural).^21^ Talking Health evaluated the effectiveness of SIPsmartER, a behavioral intervention aimed at decreasing the consumption of SSB to less than 8 fluid ounces, against a matched contact comparison group targeting physical activity called MoveMore. To be eligible, participants had to be >18 years of age, consume >200 kcal of SSB per day, have access to a telephone, and have no physical activity limitations. Recruitment was conducted using both active (e.g., direct contact with participants at health departments, clinics, and apartment complexes) and passive methods (e.g., newspaper ads, flyers, and targeted postcard mailings).^22^ This study took place between March 2012 to November 2014, and all Talking Health trial study procedures were approved by the Virginia Tech Institutional Review Board. Complete details on the study protocol and primary outcome findings are reported elsewhere.^19^,^20^

### Measures

#### Demographics

Data were collected on age, gender, race/ethnicity, income, education status, and health literacy at baseline. Age was reported on a continuous scale and recoded into four categories. Race was reported across five categories and categorized into White (non-Hispanic), Black (non-Hispanic), and other (all others). Income was reported on 12 categories, starting a <$5,000 to >$55,000 and condensed into ≤$14,999, $15,000–$34,999, and ≥$35,000. Education status was reported across six categories from no education to completion of graduate school and was collapsed into ≤ High School and ≥ Some College. Health literacy was assessed using the interviewer administered Newest Vital Sign (NVS). The NVS is a validated six-item questionnaire that assesses health literacy using a nutrition facts label. Participants can receive a score on a scale of 0–6.^23^ Using validated scoring procedures, scores for this study were collapsed to represent low health literacy (0–3) and high health literacy (4–6).

#### Dietary Intake

Dietary information was collected at baseline via three-interviewer administered nonconsecutive 24-hour dietary recalls over a 2-week period. Interviews were performed by trained researchers supervised by a PhD-level Registered Dietitian. Following the gold standard protocol for 24-hour dietary recalls,^24^ a multiple-pass method was used and recalls included for one weekend day and two weekdays. The first recall was obtained in-person at the baseline assessment and the following two were conducted through unannounced telephone calls. All of the participants completed at least one baseline 24-hour dietary recall, 90.7% completed two recalls, and 74.1% completed all three recalls. Dietary recalls were entered into a nutritional analysis software (Nutrition Data System for Research (NDS-R) 2011, University of Minnesota).

### Data Analyses

To understand demographic differences in added-sugar intake, data were extracted from NDSR and entered into SPSS 25.0 (IBM Corp., Armonk NY) for further analysis. First variables were created for total energy (kcals) and total added sugar (g) by averaging amounts from the reported number of recall days for each participant. Next, energy from added sugar was calculated by using the standard of four calories per gram of added sugar. This value was then divided by total energy (kcals) to obtain an average percentage of energy intake from added sugar. Means and standard deviations for added-sugar intake in grams and as a percentage of total energy intake were calculated. One-way ANOVAs were conducted to identify significant bivariate associations between demographics variables and total added-sugar intake and as a percentage of total energy intake. Post-hoc analyses were performed using the Tukey method. A p-value of <0.05 was used to determine statistical significance.

**Figure 1:**
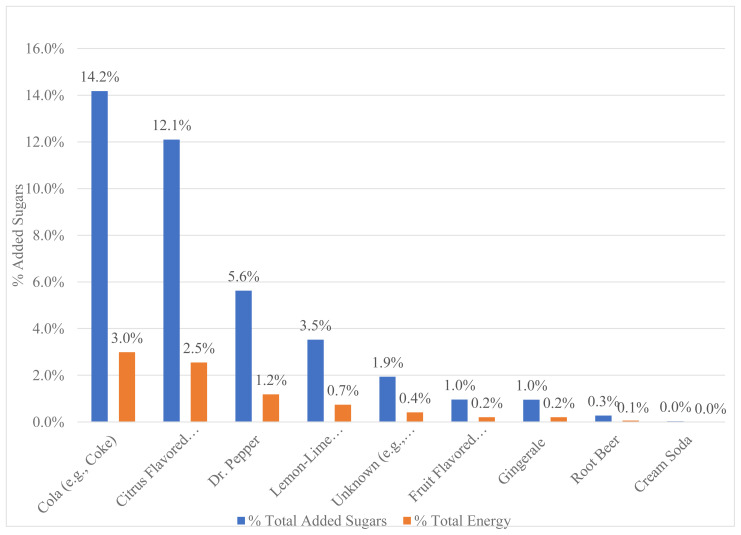
Top Drink Sources of Added Sugar: Soda

To analyze top food sources of added sugar, data were extracted from the NDSR Output File 02 (displays foods as whole versus at the ingredient level) and imported into Microsoft Excel (version 15.32). Food categories were defined using a combination of the Nutrition Coordinating Center (NCC) Food Group Identifier (numbers assigned to specific food and beverages) from the NDS-R system, and previous literature that examined dietary sources of nutrients on a national level.^25^ Some categories were collapsed (e.g., cookies, brownies, cakes were collapsed into grain desserts) or expanded (e.g. fruit flavored drinks were expanded into sports drinks and sweetened fruit drinks) for clarity of food groups that contained added sugar. Soda was further expanded by flavors (Figure 1) and analyzed.

Data were summed across all participants and all available recalls to obtain total added sugar (g) and total energy (kcals) from all foods. The NCC Food Group ID was used to identify and sort each individual food category and added sugar (g) for each of these categories was summed and reported as a proportion of total added sugar. Additionally, for each individual food category, the summed value for added sugar (g) was converted to calories using four calories per gram estimate. This total added sugar (kcals) value for each individual food category was then divided from the total energy (kcals) from all foods to obtain a proportion of total energy intake (kcals). Finally, to determine top sources of added sugar, food categories were ranked based on their added-sugar contribution to total added sugar and total energy intake.

## RESULTS

### Demographics and Differences in Added Sugar Intake

Participant demographics and differences in added-sugar intake are shown in Table 1. The majority of the sample (n=301) was female (81%), between the ages of 35 and 54 (49%), white (93%), had some college education (68%), had an income of $14,000 or less (43%), and were categorized as having high health literacy (67%).

On average, participants consumed 108.75 grams of added sugar, which contributed to around 21% of total energy intake. There were no significant differences in intake by age, race/ethnicity, education level, or health literacy status categories. However, significant differences in intake were found for the gender and income variables (p<0.05). Male participants consumed significantly more added sugar in grams when compared to females, but the amount was not significant when considering added sugar as a percentage of total energy intake. Participants who had an income greater than $35,000 consumed significantly lower amounts of total added sugar and as a percentage of total energy intake when compared to those who had an income of $14,999 or less.

### Top Sources of Added Sugars

All sources of added sugar by food and beverage categories that contributed more than 0.1% to total added sugar are ranked in Table 2. Soda was the top source of added sugar, making up almost 40% of added-sugar grams and 8% of total energy intake. Sweetened tea accounted for around 13% of added sugar and 3% of total energy intake. Following these two liquid sources, grain desserts was the third most important source, and the top solid food source of added sugar. Grain desserts, which included all types of cookies, cakes, brownies, and pies, accounted for around 7% of added sugar and 1.5% of total energy intake. Sweetened coffee and frozen dairy desserts rounded out the top five food sources.

The top liquid sources of added sugar (i.e. SSB) were soda, sweetened tea, sweetened coffee, sweetened fruit drinks (Table 2). Together all liquid sources made up around 65.5% of added-sugar foods and 13.8% of total energy intake. These proportions are about double the amount contributed by solid food sources, at 34.5% and 7.3%, respectively for added sugar and total energy intake.

### Top Sources of Added Sugars by Soda Types

Eight different soda types emerged from the analysis of dietary recalls and are shown in Figure 1. The top source contributing to added sugar from the soda food category was cola (e.g. Coke and Pepsi) at 14.2%, followed closely by citrus flavored sodas (e.g., Mountain Dew and Mello Yello) at 12.1%. Together, cola and citrus flavored sodas accounted for 26.3% of added sugar and 5.5% of total energy intake.

## IMPLICATIONS

This is the first known study to identify the top food and beverage sources of added sugar in the diets of rural Appalachian adults and to examine their relative contributions to added sugar and energy intake. This study is an important step to help inform interventions and policies targeting added-sugar behaviors in this health disparate region. These findings also reinforce the need to reduce to overall added-sugar intake in rural diets, particularly in males and lower-income rural residents. This can be achieved through reducing consumption of SSB, due the high proportion consumed relative to other food sources. Therefore, efforts that aim to reduce added-sugar intake and that strive to make significant impacts on rural health should focus on SSB intake, with a strong message to reduce sodas.

These results should be interpreted within the context that consuming greater than 200 calories of SSB per day was one of the inclusion criteria for the Talking Health trial.^19^ Therefore, when trying to compare findings to other national and regional studies with no SSB intake inclusion criteria, the percent of total added-sugar intake and percent of energy from added sugar from the current study may be inflated. However, it is important to note, a different cross-sectional study in this same southwest Virginia study region, but with no SSB inclusion criteria, found a remarkably similar average SSB intake (i.e., 457 kcals/day). This same study found that 82% of respondents exceeded the SSB recommendation of less than 8 ounces per day and corroborates that SSB intake in this region far exceed national averages of SSB intake (i.e., 138 kcals/day).^17^,^18^,^26^ Also, when comparing this study demographics to the U.S. Census data in the targeted Appalachian region, this sample was representative in terms of age, education, and race (i.e. 94% vs. 93% Whites).^22^ Nonetheless, the limited racial diversity and under representation of men (i.e., 18.6%) in this sample should be considered when interpreting the study findings.

The intake of added sugar in this sample is more than double the recommended amounts by the 2015–2020 Dietary Guidelines for Americans and World Health Organization (10% of energy intake), and can be cautiously compared to the national estimate of 13% of added sugar from total energy intake.^8^ In general, findings pertaining to differences in added-sugar intake by gender and income align by findings from nationally representative samples^27^; therefore, future recruitment strategies should target these specific subgroups and increase their enrollment, engagement and retention in interventions that reduce added-sugar intake. For example, analysis of the recruitment methods in the Talking Health trial found that using more active recruitments yielded more male participants compared to passive strategies and was also more successful at recruiting lower income participants.^22^

Even without considering other added-sugar sources, SSB contribution to total energy intake in this sample was 40% higher than the recommendations for total added sugar.^6^ When looking at relative contributions, the substantially higher quantities of SSB compared to solid sources, in this rural sample is especially concerning. Excessive SSB intake is the only known added-sugar source that has been linked to weight gain and diseases such as Type-2 diabetes and heart disease.^28^ As SSB contributes to more than half of added-sugar intake in this rural population, reducing the consumption of this single food group may have significant health implications.

The high soda intake is also troublesome in Appalachia due to the potential link between soda acidity and oral health disparities in this region.^29^ Krause and colleagues reported that compared to non-Appalachian residents, more Appalachian adults had reported that they had six or more teeth removed as a result of preventable causes (12.9% vs. 10.9%).^30^ In Appalachia this poor oral health crisis has been referred to as “Mountain Dew Mouth” due to the idea that citrus-flavored sodas are the most highly consumed.^29^ This study identified cola as the top contributor followed closely by citrus (e.g., Mountain Dew) flavored drinks. Reddy and colleagues found that most cola and citrus flavored sodas had pH levels below 4.0 and categorized them in the “extremely erosive” and “erosive” categories.^31^ While reducing added-sugar intake via reductions in soda consumption is an important target for interventions improving rural health, it is also important that health messages convey the importance of being mindful of the acidity level of replacement beverages (e.g., diet version can also be acidic).

This study highlights potential opportunities and barriers for local and regional public policies in rural regions that focus of reducing the consumption of SSB. For example, rural policy makers could consider a concerted effort that includes imposing regulations on advertisement of SSB and soda and implementing a broad education and marketing campaign that focuses on reducing soda, using images of culturally relevant beverages such as cola and citrus flavors. Another suggested strategy is implementing SSB taxes. Several U.S. jurisdictions (e.g. Berkeley, Philadelphia, San Francisco, Oakland, Albany, Boulder, and Cook County in Illinois) have enacted SSB taxes.^32^ However, SSB taxes are highly controversial and have been suggested to be regressive, particularly for low-income communities.^33^ For example, advocates for taxation have struggled to garner support in largely rural areas, including Appalachian regions.^34^ Other documented reasons for higher SSB intake in rural and low socioeconomic U.S. subgroups include factors such as the relative low cost of SSB, high SSB availability, concerns with drinking water quality, industry targeted SSB marketing strategies, lack of awareness pertaining to SSB risks, and lack of effective SSB reduction interventions and policies among these populations.^35^ Therefore, taxation and other local policy level strategies should be considered within the cultural context of the numerous factors driving high added-sugar consumption patterns. In conjunction with these previously published findings, results from this study may be applied to further understand the potential positive and negative impacts of SSB polices and taxation strategies in the Appalachian region.

In addition to the 200 calories of SSB per day inclusion criteria for this study and the restrictions this imposes to interpreting the findings relative to national estimates, several other limitations should be considered. First, this study used a non-probability sampling approach, may reflect self-selection bias, and may have limited generalizability to other populations beyond the Appalachian region of rural southwest Virginia; yet can be used to guide similar studies in other regions. Similarly, this sample was representative for the region,^19^ with the exception of men, which may also limit generalizability. Lastly, self-reported dietary recalls are prone to measurement error.^36^ However, one of the strengths of this study was the use of multiple pass methods along with three dietary recalls obtained separately, that included two weekdays and one weekend day, for a more comprehensive representation of dietary intake.^24^

Results from this study can be used to inform and modify nutritional messages in these adapted interventions, to increase cultural relevancy and potentially the effectiveness of the intervention. To further understand this, future studies should explore how specific food and beverages choices changed as a result of incorporating specific food-based dietary recommendations. Future research should also aim to obtain samples with greater diversity to help understand the differences in intake of added sugar within these demographic subsets.

**SUMMARY BOX**

**What is already known on this topic?**

Rural residents have high consumption of added sugars and sugar-sweetened beverage that contribute to nutrition-related health disparities. In rural Appalachia, added-sugar intake contributes to around 21% of total energy intake which is 7% higher than national intake and two times more than the recommended amounts

**What is added by this report?**

In this sample of high sugar-sweetened beverage consuming participants, males and those with lower income consumed more added sugar. Liquid sources contributed two times more than solid sources to energy intake. The top source was soda, with cola and citrus emerging as the top flavors.

**What are the implications for future research?**

This study can be used to help prioritize interventions and policies that are focused on the foods and beverages consumed most often in rural populations.

## Figures and Tables

**Table 1:** Participant Demographics and Differences in Added Sugar Intake and Added Sugars As a Percentage of Total Energy (N=301)

	n (%)	Added Sugars (g) Mean (SD)	F-Statistic (p-value)	% Total Energy Mean (SC)	F- Statistic (p-value)
**Gender**					
Male	56 (18.6)	120.7 (89.7)	4.10 (0.04)	19.5 (11.5)	1.41 (0.24)
Female	245 (81.4)	96.8 (77.0)		21.6 (11.8)	
**Age (years)**					
18–34	96 (31.9)	109.8 (95.0)	0.79 (0.50)	22.1 (12.1)	0.30 (0.82)
35–54	148 (49.2)	99.8 (75.8)		20.9 (12.1)	
55–64	50 (16.6)	92.6 (62.0)		20.9 (10.6)	
65+	7 (2.3)	77.3 (40.1)		19.0 (6.7)	
**Race/Ethnicity**					
White (non-Hispanic)	280 (93.0)	102.3 (81.0)	0.32 (0.73)	21.4 (11.9)	0.87 (0.42)
Black (non-Hispanic)	13 (4.3)	87.4 (38.5)		20.9 (6.7)	
Other	8 (2.7)	88.7 (94.5)		15.8 (13.6)	
**Education Level**					
≤High School	96 (31.9)	101.4 (79.6)	0.00 (0.98)	21.9 (12.4)	0.54 (0.46)
≥Some College	205 (68.1)	101.2 (80.2)		20.9 (11.4)	
**Income Category**					
≤$14,999	129 (42.9)	113.2 (101.3)*	4.45 (0.01)	23.7 (14.2)*	7.58 (0.00)
$15,000–$34,999	96 (31.9)	102.6 (63.2)*,†		21.0 (9.2)*,†	
≥$35,000	76 (25.2)	79.3 (47.1)†		17.2 (8.6)†	
**Health Literacy (NVS)** §					
Low	99 (32.9)	96.0 (76.9)	0.64 (0.43)	21.2 (12.7)	0.00 (0.98)
High	202 (67.1)	103.8 (81.4)		21.2 (11.3)	

*, † Post-hoc analyses were done using the Tukey method. Values that do not share the same symbol are significantly different from each other (p<0.05)

§ Newest Vital Sign (NVS) is a validated six-item questionnaire that assesses health literacy using a nutrition facts label. Participants receive a score on a scale of 0–6. Scores were collapsed to represent low health literacy (0–3) and high health literacy (4–6).

**Table 2:** Top Sources of Added Sugars by Food and Beverage Categories

Rank	Food and Beverage Category	Liquid or Solid	% Total Added Sugars*	% Total Energy
1	Soda (e.g., cola, citrus flavored, root beer)	Liquid	39.6	8.3
2	Sweetened tea (e.g., sweet tea, hot tea with sugar packets)	Liquid	12.9	2.7
3	Grain desserts (e.g., cookies, cakes, brownies)	Solid	6.9	1.5
4	Sweetened coffee (e.g., with cream and/or sugar)	Liquid	5.7	1.2
5	Frozen dairy desserts (e.g., ice cream, milkshakes)	Solid	4.6	1.0
6	Candy (e.g., chocolates, jellybeans)	Solid	4.1	0.9
7	Additions (e.g., dressings, spreads, or toppings)	Solid	4.0	0.9
8	Sweetened fruit drinks (e.g., lemonade, fruit punch)	Liquid	3.0	0.6
9	Cold cereal (e.g., Frosted Flakes)	Solid	2.6	0.6
10	Mixed meat entrees (e.g., meatloaf, chicken parmigiana, fast food)	Solid	2.3	0.5
11	Muffins, pastries, and quick breads (e.g., donuts, Pop-tarts, cornbread)	Solid	2.2	0.5
12	All other breads (e.g., white bread, buns, biscuits)	Solid	1.8	0.4
13	Sports drinks (e.g., Powerade, Gatorade)	Liquid	1.8	0.4
14	Yogurt (e.g., strawberry yogurt)	Solid	1.2	0.3
15	Energy drinks (e.g., Redbull, Monster)	Liquid	1.2	0.2
16	Snacks (e.g., chips, popcorn, pretzels, granola bars)	Solid	1.0	0.2
17	Hot cereal (e.g., brown sugar oatmeal)	Solid	0.8	0.2
18	Sweetened milk (e.g., chocolate milk)	Liquid	0.8	0.2
19	Fruits (e.g., canned fruits)	Solid	0.6	0.1
20	Other desserts (e.g., pudding, Jell-O)	Solid	0.5	0.1
21	Frozen non-dairy desserts (e.g., popsicles)	Solid	0.5	0.1
22	Specially formulated drinks (e.g., Carnation Instant Breakfast)	Liquid	0.4	0.1
23	Mixed vegetable dishes (e.g., salads, coleslaws)	Solid	0.3	0.1
24	Processed meats (e.g., hot dogs, lunchmeats)	Solid	0.3	0.1
25	Beans (e.g., canned baked beans)	Solid	0.2	0.0
26	Mixed grain dishes (e.g., pizza w/out meat, peanut butter and jelly)	Solid	0.1	0.0
27	Breakfast grains (e.g., pancakes, French toast)	Solid	0.1	0.0
28	Soup (e.g., ramen noodle)	Solid	0.1	0.0
29	Cocktails (e.g., wine coolers)	Liquid	0.1	0.0

* Only foods that contribute to ≥0.1% of added sugars are presented in the table.
